# Predicting Parental Mental Health During COVID-19: Economic Pressure, COVID-19 Stress, and Coping Strategies

**DOI:** 10.3389/fpsyg.2022.909978

**Published:** 2022-07-22

**Authors:** Derek Daniel Morgan, Connað Dael Higgins, Paul B. Ingram, Christy Rae Rogers

**Affiliations:** ^1^Department of Human Development and Family Sciences, Texas Tech University, Lubbock, TX, United States; ^2^Department of Psychological Sciences, Texas Tech University, Lubbock, TX, United States

**Keywords:** parents, COVID-19, coping, anxiety, depression

## Abstract

As the COVID-19 pandemic continues, understanding connections between economic pressures and mental health experiences is critical in comprehending how stressful global events can affect families. Although economic pressures and stress can negatively impact mental health, approach coping strategies may provide reductions in negative mental health experiences for parents compared to avoidant coping strategies. Despite recent work showing that stress resulting from the pandemic can have negative implications for the mental health of parents with young children, there is little known about the mental health of parents with adolescents. This study utilized a longitudinal sample of 198 parents (194 biological parents; 103 Fathers, and 91 Mothers) of adolescents and examined the mediating impact of COVID-19 stress on the relationship between economic pressure and subsequent depressive and anxious symptoms. Additionally, approach and avoidant coping strategies were examined as potential moderators between COVID-19 stress and later mental health. Results indicated that parents who experienced economic pressure reported worsening mental health across the school semester, with COVID-19 stress mediating this pathway. Further, approach coping strategies moderated the association between COVID-19 stress and later anxiety symptoms such that higher levels of coping associated with greater rates of later anxiety symptoms, while lower levels of coping associated with less anxiety symptoms later. Avoidant coping strategies also moderated these associations, such that greater use associated with greater depressive and anxious symptomology later. These findings emphasize that parents are experiencing worsening mental health following the onset of the pandemic and that there is an urgent need for increased mental health services to assist families during this time.

## Introduction

The coronavirus (COVID-19) was declared a pandemic in early 2020 by the World Health Organization (Centers for Disease Control and Prevention, 2021). In addition to its ongoing health impacts, COVID-19 continues causing global restrictions on travel (National Center for Immunization and Respiratory Diseases, 2021), requiring country-wide lock-downs and restrictions to control the spread of the virus (Viner et al., 2020). COVID-19 has also presented numerous novel social challenges for families. Social isolation has increased because of the risk of infection. Concurrently, there has been fluctuations in socio-educational environments for families. Accordingly, mental health in families has been affected (Calvano et al., 2021; Westrupp et al., 2021). Parents of school aged children reported increased stress and depression during pandemic-related school closures in 2020, resulting in online education in the hopes of preventing the spread of COVID-19 among youth (Bäuerle et al., 2020; Jeffs et al., 2020; Lee, 2020; Viner et al., 2020). However, switching from in-person to online education left parents with the burden of managing their children’s education while also working from home. As the pandemic continues its disruptive course, understanding connections between these economic pressures and mental health experiences is critical in understanding how stressful global events can affect families. The family stress model (FSM; Conger et al., 2002, 2010) offers a framework to contextualize links between economic strains and worsening mental health in the context of COVID-19, particularly as there is little research linking these pathways in parents (Kämpfen et al., 2020), and less even on families (Prime et al., 2020; Wahlund et al., 2020).

### COVID-19 and Mental Health

Examining the effects of COVID-19 on family function is paramount to understanding mental health within the family context. Given that environmental and personal stressors influence mental health (e.g., Hogan, 2003; Conger et al., 2010), the pandemic presents a singular stressor that has influenced life broadly. Early studies of the pandemic’s impact paint a dim view of family adaptability to COVID-19, as parents and children report increased stress and depressive experiences (Ho et al., 2020; Jeffs et al., 2020; Usher et al., 2020). Likewise, negative effects of COVID-19 are similarly documented for parental adaptability to stress (Jeffs et al., 2020; Viner et al., 2020; Kar et al., 2021) and parental emotional wellbeing (Wilson et al., 2020; Lee et al., 2021). The ability to manage worry during the pandemic has dwindled worldwide as parents report greater levels of anxiety (Ho et al., 2020; Lee et al., 2021).

In recent studies, the percentage of parents who experience moderate to high anxiety during the pandemic are exceptionally high, up to 20% or more (Barzilay et al., 2020; Wahlund et al., 2020; Wilson et al., 2020; Kar et al., 2021), compared to the normative adult average of approximately 5.6% (Brown et al., 2001; SAMHSA, 2020, 2021). Parents and caregivers of school aged children experienced increased anxiety during the initial months of COVID-19 (Barzilay et al., 2020; Wu et al., 2020), along with increased worry regarding COVID-19 (Bäuerle et al., 2020; Steele, 2020). As with anxiety, rates of depression (e.g., feeling sad, lethargic, apathetic, demoralized, or hopeless) during COVID-19 have increased dramatically. Indeed, research has found that up to an alarming 40% of parents (compared to the normative adult baseline of 9%) report significant depressive symptoms culminating in clinically impactful levels of impairment (SAMHSA, 2020, 2021; Lee et al., 2021). Parents of young children during COVID-19 experience even higher depressive symptoms (Wu et al., 2020; Lee et al., 2021) than those with older children, such as adolescents. Even so, parents of adolescents continue to navigate depression on a daily basis (National Research Council and Institute of Medicine, 2009).

As children transition into the period of adolescence and develop more independence, parents are less knowledgeable about their child’s behaviors and whereabouts, which can result in increased stress for parents (Small et al., 1988). Adolescents navigate greater autonomy, less supervision and less direct care compared to young children (Noom et al., 2001). Parents also report less understanding of their adolescent’s thinking, which often associates with communication disparities and conflict in parent-adolescent relationships (Sillars et al., 2005). Given that parents of adolescents often navigate increased stressors related to their adolescent’s typical development, increased stress from the pandemic may compound these normative stressors and worsen their overall mental health. Yet, few studies have examined the mental health of parents of adolescents, despite their role in navigating more complex interactions with their increasingly autonomous children (Viner et al., 2020; Wu et al., 2020). In short, understanding family functioning during the COVID-19 pandemic requires consideration of the ubiquitously emotional and mental health states.

As the COVID-19 pandemic has progressed, resources that were available prior to the pandemic’s onset have become more sparse. Indeed, psychological services, accounting for the emergency telehealth allocation for regional providers, have become less available as the needs of new and current clients have increased (Turabian, 2020; McLean and McIntosh, 2021). Furthermore, practitioners addressed client needs alongside their own, often at personal sacrifice (McLean and McIntosh, 2021). With the limited understanding of the COVID-19 virus, many practitioners were unprepared for shifts in service modality (i.e., in-person to virtual), and the mental health ramifications of lockdowns enforced by local governments.

### Parenting Stressors: Economic Pressure and COVID-19

Millions of families across the globe have been affected by economic difficulties due to pandemic restrictions on travel, reduced employment opportunities, and lost jobs. Unsurprisingly, the early stages of the pandemic witnessed the greatest peak in unemployment, prompting many countries to enact higher unemployment benefits and stimulus payments to their citizens (Kämpfen et al., 2020; Wilson et al., 2020). Economic pressure resulting from the pandemic has affected the ability of families to purchase the resources they need to survive, such as food, utilities, and housing. This impact on resource availability, in turn, initiates a cascade of economic problems (e.g., Fallon et al., 2020), which often leaves the mental health of family members neglected in the process (Conger et al., 2002, 2010). Mental health problems caused or exacerbated by the pandemic must be addressed (Barzilay et al., 2020; Bäuerle et al., 2020; Wu et al., 2020) as the mental health of family members may be a looming crisis due to the impact of isolation and excessive worry caused by the pandemic (Galea et al., 2020; Kathirvel, 2020). Thus, examining mental health alongside economic pressure is a logical step in contributing to a contextualized understanding of mental health in families, and how to best address these issues in mental health practice.

The FSM proposes that economic pressure has broad and potentially negative effects on parent mental health (Conger et al., 2002, 2010). Increased economic pressure exacerbates problematic psychological functioning and worsens parental anxiety and depression (Masarik and Conger, 2017). Thus, families are at higher risk of developing worsening mental health when they experience economic pressure (Prime et al., 2020), with low income families experiencing more mental health problems than those with greater income (Evans et al., 2020). However, the contextual factors that may explain the transmission of stress from economic pressure to mental health functioning is less understood.

Given the salience of negative life events on economic pressure and parent mental health (Masarik and Conger, 2017), it is critical to identify timely societal stressors that may explain this association. As economic burdens, such as job loss, can have detrimental impacts on adult mental health in a relatively short period (Burgard et al., 2007), it is essential to have timely reports of mental health functioning close to that of economic and stressful events. With the announcement of the pandemic, parents reported increased stress during the initial months of COVID-19 and the subsequent academic school year (Sonnenschein et al., 2021). The combination of economic stress and parenting stress during COVID-19 can influence the mental health of families in relatively short time spans, as suggested in prior research (Evans et al., 2020; Dvorsky et al., 2021). As families attempt to address the increased stressors affecting their daily lives, it becomes essential to gather proximal timely reporting of family functioning to understand changes in family mental health. The FSM emphasizes the importance of event conceptualization of stressful life events. The way in which an individual perceives chronic stressors can affect their mental health (Lu, 1994). Additionally, the FSM proposes that protective factors (i.e., coping strategies) can moderate the association between perceptions of stressful life events and their later mental health. Examining the interaction of event conceptualization (e.g., COVID-19 stress) and protective factors (e.g., coping strategies) then may predict later mental health, as one’s mental health is influenced by their environment and interactions with it.

### Coping Strategies

The FSM proposes individual protective factors, such as coping strategies, can provide an avenue to reduce the effects of economic hardship on mental health. Coping strategies are known to reduce stressful and depressive experiences (Kim et al., 2008; Kadhiravan and Kumar, 2012), and can be conceptualized as either approach (i.e., strategies for adapting thoughts or actions to a given situation) or avoidant (i.e., behaviors consistent with ignoring or dissociating from given situations; Folkman and Lazarus, 1988). Approach coping strategies are more effective in dealing with stressful events than avoidant coping (Babore et al., 2020), as approach coping changes the way individuals interact with their environment, while avoidant coping provides distractions and redirects to avoid confronting and addressing stressful events. Approach coping adapts an individual’s behavior and encourages healthy management of feelings and emotions (Folkman and Lazarus, 1988). Unfortunately, effective use of approach coping during periods of high stress often requires instruction, such as resources that are provided in clinical settings (Ergüner-Tekinalp and Akkök, 2004; Babore et al., 2020). However, avoidant coping, which reinforces a disregard for current stressful experiences, are readily available and easily learned, such as with various substance use (Tate et al., 2006). During times of crisis, such as the pandemic, approach coping strategies have been utilized at a lower rate than avoidant coping among adults, including parents (Verger et al., 2020; Kar et al., 2021). Reductions in anxious and depressive symptoms, along with increases in self-worth and esteem, have been found when parents employ approach coping skills compared to avoidant (Kadhiravan and Kumar, 2012; Babore et al., 2020). To date, the interplay between coping strategies and stress from COVID-19 has not been examined in parental populations. Additionally, studies on parents of adolescents are particularly needed given the substantial and unique stress experienced within these households.

## Current Study

This study used a longitudinal sample of parents and caregivers of adolescent middle school aged children to examine mental health during the first in-person school semester following the COVID-19 pandemic. Expanding on the FSM framework, this work aimed to elucidate the mechanisms of COVID-19 stress between economic pressure and mental health, and coping strategies as a potential buffer between COVID-19 stress and mental health (Masarik and Conger, 2017). We hypothesized that (a) higher economic pressure would positively predict later depression and anxiety symptoms, (b) COVID-19 stress would mediate the association between economic pressure and later depression and anxiety, such that higher COVID-19 stress would positively predict later depression and anxiety, (c) approach coping strategies would moderate the association between COVID-19 stress and later mental health symptoms (e.g., anxiety and depression), such that greater use of approach coping strategies would diminish the positive association between COVID-19 stress and later mental health, and (d) avoidant coping strategies would moderate the association between COVID-19 stress and later mental health symptoms, such that greater use of avoidant coping strategies would exacerbate the positive association between COVID-19 stress and later mental health.

## Materials and Methods

### Participants

This study used longitudinal data from the Resilient Families Study, gathered from parents and caregivers of 7 or 8th grade adolescents residing in Texas. To maximize inclusion of various family types, parents, and caregivers (herein referred to as parents), included biological, step, and adoptive parents, as well as aunts and uncles as these individuals may provide significant caregiving for adolescents. All participants reported providing primary care for the adolescent in a parental role. Of the 212 participants who completed the first online survey, 14 were removed based on data quality recommendations (e.g., slow response time, inconsistent demographics; Teitcher et al., 2015). Specifically, we removed individuals from analyses if they had identical IP addresses, unusually fast survey completion rate (i.e., completion time under 10 min), or inconsistent responses. The final sample of 198 participants (*M*_age_ = 42.0 years old, range = 30–56 years old; 94 females) identified primarily as White (92.1%) and reported being a biological parent (*n* = 194; 103 Fathers, 91 Mothers), stepmother (*n* = 1), aunt (*n* = 1), uncle (*n* = 1), or adoptive mother (*n* = 1) of an adolescent child. Parents reported on 198 adolescents between 7 and 8th grade for this study (*M*_age_ = 14.22 years old, range = 9–16 years old; 68 female). As shown in Table 1, most parents earned an annual household income between $45,000 and $74,999 (64.7%), completed at least some college (95.4%), and were married (93.9%).

**Table 1:** Demographics: ethnicity, family total income, parental education, and marital status.

Variables	*N* (%)
Parental ethnicity	
Caucasian/White	182 (91.91%)
Hispanic/Latino	6 (3.03%)
Caribbean	6 (3.03%)
Multiethnic	4 (2.02%)
Family total income (*N* = 197)	
<$45,000	19 (9.6%)
$45,000–$74,999	128 (64.7%)
$75,000–$99,999	39 (19.7%)
$100,000–$150,000	6 (3.0%)
> $150,000	5 (2.5%)
Parental education (*N* = 198)	
Some high school	1 (0.5%)
High school diploma	8 (4.0%)
Some college	47 (23.7%)
Associate’s degree	49 (24.7%)
Bachelor’s degree	62 (31.3)
Some graduate school	19 (9.6%)
Master’s degree (e.g., M.A. and M.S.)	11 (5.6%)
Professional degree (e.g., M.D. and Ph.D.)	1 (0.5%)
Marital status (*N* = 198)	
Single (never married)	5 (2.5%)
Married (first, second, and third)	186 (93.9)
Divorced	5 (2.5%)
Separated	1 (0.5%)

### Research Design

Eligible families were recruited through social media (i.e., Facebook) posts in local parenting groups. Participants completed a brief online questionnaire that screened for the study criteria of being a local parent/caregiver of a 7 or 8th grade adolescent who was willing to participate in two online surveys. Consent was obtained from participants through Qualtrics, in accordance with the Institutional Review Board. Eligible participants were emailed information about the study and provided a personal Qualtrics link to the first of two online surveys, which was completed at the beginning of the 2020 fall school semester (*N* = 198). The second survey was administered approximately 2 months later, near the end of the 2020 fall semester (*N* = 187; 94% retention). As parents were facing multiple stressors from the pandemic and concerns over management of the academic school year, an opportunity became apparent to understand influences on parental mental health experiences during this salient period of back to in-person schooling. As such, our surveys were administered over the fall 2020 academic semester to capture parental mental health experiences during this crucial period. At the beginning and end of each survey mental health resources were provided to participants given that some questionnaires pertained to psychological functioning. Participants were compensated with an Amazon.com gift card after completion of each survey; $10 for the first survey, $15 for the second.

### Measures

The following questionnaires were reported by parents at Time 1 (T1) and Time 2 (T2). Parents completed identical survey items at T1 and T2 for all measures reported. The time at which measures were used for the mediation models are noted in each section.

#### T1 Economic Pressure

Parents reported on their experiences of economic pressure and ability to meet economic needs over the past 3 months using the Economic Pressure Scale (Conger et al., 2002). Parents responded to nine items, the first asked about difficulty meeting economic needs with responses ranging from 0 (*no difficulty at all*) to 3 (*a great deal of difficulty*). The second asked about the family’s financial position at the end of each month, in which responses ranged from 0 (*with more than enough money left over*) to 3 (*very short of money*). Lastly, seven reverse scored items asked if parents had enough money to meet their needs in different areas (e.g., *to afford the kind of home they needed* or *to afford the utilities they needed*). Responses ranged from 0 (*not at all true*) to 3 (*very true*). Responses were summed with higher scores indicating greater economic pressure and the scale demonstrated great reliability (*α* = 0.87).

#### T1 COVID-19 Stress

Parents reported their experiences of stress related to COVID-19 using the COVID-19 Stress Scale (Ladouceur, 2020). This scale examines the effects of COVID-19 on adolescents and through parental report, and research has supported their effectiveness in doing so (Dvorsky et al., 2021; Styck et al., 2021). Parents rated their stress about COVID-19 on four items asking, “How worried have you been about…” including, “Your physical health being influenced by COVID-19,” and, “Friends or family being infected,” with scores ranging from 0 (*not at all*) to 5 (*extremely*). Responses were summed with higher overall scores indicating greater stress related to COVID-19 and the scale yielded excellent reliability (*α* = 0.90).

#### T1 Coping Strategies

Parents completed the Brief COPE (Carver, 1997), which has been used to examine adaptive (approach) and maladaptive (avoidant) types of coping strategies. The Brief COPE is comprised of 28 items that make up 14 subscales (two items each). Seven subscales represent approach coping strategies and seven represent maladaptive coping strategies (for review, see Carver, 1997). For this study, we examined approach coping strategies given their association with adult well-being (Carver, 1997; Verger et al., 2020) and our aim to identify a potential buffer against the effect of COVID-19 stress and poor parent mental health. We additionally examined avoidant coping strategies to further compare potential moderating effects between approach and avoidant coping.

We used six approach coping subscales (active coping, instrumental support, planning, acceptance, emotional support, and positive reframing) that included two items each. Religious coping was excluded as prior studies have indicated that this type of coping can associate more with maladaptive strategies, rather than with approach strategies (Krägeloh, 2011). Example items included, “I’ve been trying to come up with a strategy about what to do,” and “I’ve been looking for something good in what is happening,” and ranged from 0 (*I have not been doing this at all*) to 3 (*I’ve been doing this a lot*). To create the approach coping strategies scale, six approach subscales were averaged and yielded satisfactory reliability (*α* = 0.81), with higher scores indicating greater use of approach coping strategies.

To measure avoidant coping, we used six of the maladaptive coping subscales (behavioral disengagement, denial, self-distraction, self-blame, substance use, and venting) comprised of two items each. Humor coping was excluded due to its adaptability for both approach and avoidant coping styles (Dozois et al., 2009). Items such as “I’ve been refusing to believe that it has happened,” ranged from 0 (*I have not been doing this at all*) to 3 (*I’ve been doing this a lot*). Subscales were averaged to create the avoidant coping strategies scale, which yielded satisfactory reliability (*α* = 0.86), with greater scores indicating greater utilization of avoidant coping.

#### T2 Depression

Parents reported on their depressive symptoms using the Patient Health Questionnaire 9-Item (PHQ9; Kroenke et al., 2001), which is a widely used depression screening measure that is often incorporated into patient-based care and clinicial treatment evaluation. Items evaluate depressive symptom experiences over the past 2 weeks and align with the diagnostic criteria of a depressive episode (American Psychiatric Association, 2013) with individual response scores ranging from 0 (*not at all*) to 3 (*daily*). Response items were summed with higher overall scores indicating higher depressive experiences and the scale revealed high internal consistency (*α* = 0.85).

#### T2 Anxiety

Parents reported on their anxiety symptoms using the Generalized Anxiety Disorder 7-Item (GAD7; Spitzer et al., 2006b), which is a widely used measure of anxiety symptoms and internalized metal health distress. Items are consistent with the diagnostic criteria for generalized anxiety (American Psychiatric Association, 2013), with responses ranging from 0 (*not at all*) to 3 (*daily*). Items are summed to produce a total score, with higher scores indicating greater anxiety symptoms. The GAD7 scale had high internal consistency (*α* = 0.84).

#### T1 Covariates

Participants reported on their gender, household income last year (2019), highest level of education, and personal distress, which were included as covariates given their association with the variables of interest. The personal distress subscale from the Brief Interpersonal Reactivity Index (Davis, 1983) was used to assess parental feelings of personal anxiety and unease in tense interpersonal settings. We controlled for personal distress in the model as it gauges individual-level of stress in given situations, and it allowed for a more robust integration of COVID-19 stress outside of normal levels of distress individuals feel in everyday life. The personal distress subscale is made up of seven items scored from 0 (*does not describe me well*) to 4 (*describes me well*). Response items were summed with higher scores indicating greater personal distress; internal consistency was acceptable (*α* = 0.70).

### Analysis Plan

Analyses were conducted in SPSS version 27 (IBM Corp. 2020) using the PROCESS macro (Hayes, 2013). The PROCESS macro provides models to test mediation and moderated mediation, both of which were utilized with outcome variables depression and anxiety. The mediation models examined COVID-19 stress as a mediator between economic pressure and the outcomes depression and anxiety. The second set of models examined moderated mediation of the previous models with approach and avoidant coping strategies as a moderator between COVID-19 stress and the outcome variables of depression and anxiety. Covariates for all models included last year’s household income, and parental education, gender, and initial levels of personal distress. The data that support the findings of this study are available upon request from the corresponding author.

**Figure 1:**
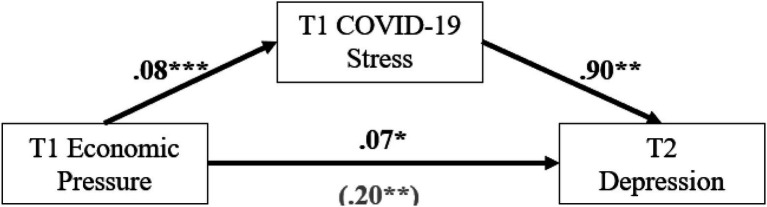
COVID-19 stress as a mediator between economic pressure and later depression. *p < 0.05; **p < 0.01; ***p < 0.001.

**Figure 2:**
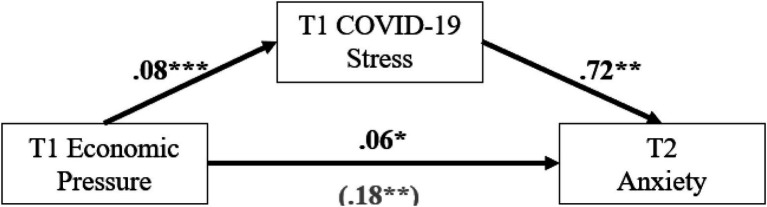
COVID-19 stress as a mediator between economic pressure and later anxiety. *p < 0.05; **p < 0.01; ***p < 0.001.

**Figure 3:**
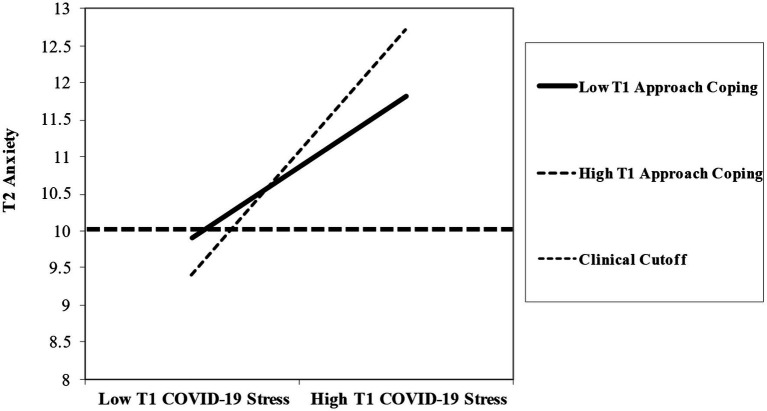
Interaction between COVID-19 stress and approach coping predicting later anxiety.

**Figure 4:**
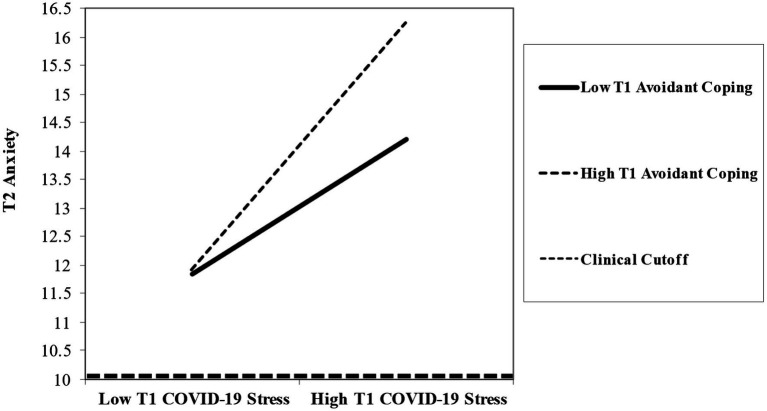
Interaction between COVID-19 stress and avoidant coping predicting later anxiety.

## Results

### Descriptive Statistics

Bivariate correlations, means, and SDs of study variables are presented in Table 2. Economic pressure at T1 was significantly and positively correlated with T1 COVID-19 stress (*r* = 0.32), T1 approach coping (*r* = 0.16), T2 anxiety symptoms (*r* = 0.25), and T2 depressive symptoms (*r* = 0.29), such that higher economic pressure associated with higher levels of all other variables. Personal distress was significantly and positively correlated with T1 avoidant coping (*r* = 0.56), T2 anxiety symptoms (*r* = 0.39), and T2 depressive symptoms (*r* = 0.44), but not with T1 COVID-19 stress (*r* = 0.05) or T1 approach coping (*r* = 0.02). Approach coping at T1 was significantly and positively associated with T1 avoidant coping (*r* = 0.45), T2 anxiety symptoms (*r* = 0.18), and T2 depressive symptoms (*r* = 0.24). Avoidant coping at T1 was additionally significantly and positively associated with T2 anxiety symptoms (*r* = 0.48), and T2 depressive symptoms (*r* = 0.52). Finally, depression and anxiety symptoms at T2 were significantly and positively correlated with one another (*r* = 0.89).

**Table 2:** Bivariate correlations and descriptive statistics of covariates and variables of interest.

Measure	1	2	3	4	5	6	7
1. T1 Economic Pressure	—	0.04	0.32^**^	0.16^*^	0.13	0.25^**^	0.29^**^	
2. T1 Personal Distress		—	0.05	0.02	0.56^**^	0.39^**^	0.44^**^	
3. T1 COVID-19 Stress			—	0.58^**^	0.33^**^	0.23^**^	0.21^**^	
4. T1 Approach Coping				—	0.45^**^	0.18^*^	0.24^**^	
5. T1 Avoidant Coping					—	0.48^**^	0.52^**^	
6. T2 Anxiety (GAD7)						—	0.89^**^	
7. T2 Depression (PHQ9)							—	
*M*	25.29	12.56	4.59	5.63	4.83	8.99	10.15	
*SD*	5.97	2.89	1.17	1.03	1.20	4.89	9.02	

M, Mean; SD, standard deviation; T1, Time 1; and T2, Time 2.

* *p* < 0.05;

** *p* < 0.01.

All correlations tested at this level. Two-tailed significance.

Parental depression symptoms at T1 (*M* = 10.01, *SD* = 5.10) and T2 (*M* = 10.15, *SD* = 6.02) were not significantly different [*t*(185) = −0.248, *p* = 0.804], indicating that parental depression symptoms did not significantly change between T1 and T2 (*d* = 0.03). As expected, depressive symptoms at T1 and T2 were significantly correlated with each other [*r*(185) = 0.492, *p* < 0.001]. Parental depressive symptoms were estimated to clinical ratings of depression at or above a score of 10 (Kroenke et al., 2001). At T1, 106 (53.8%) parents reported depressive syptoms at or above clinical cutoffs, compared to T2, 92 (49.7%) parents reported depressive syptoms at or above clinical cutoffs. Additionally, 71 (66.9%) parents maintained clinical levels of depressive symptomology from T1 to T2, with 35 parents reporting lessoning symptoms, and 21 parents reporting increases in depressive symtomology.

Parental anxiety symptoms reported at T1 (*M* = 8.99, *SD* = 4.89) and T2 (*M* = 8.08, *SD* = 4.68), [*t*(185) = 2.76, *p* = 0.006] differed statistically, but not at a clinically meaningful level (*d* = 0.19). Parental anxiety scores at T1 were significantly correlated with scores at T2 [*r*(185) = 0.575, *p* < 0.001]. Clinical cutoffs for parental anxiety symptoms were determined using recommendations from Spitzer et al. (2006b), where scores of 10 or more were indicative of clinically significant levels of anxiety symptoms likely to impair function. At T1, 86 (43.6%) parents reported anxiety symptoms at or above clinical cutoffs, compared to T2, where 77 (41.6%) parents reported anxiety symptoms at or above clinical cutoffs. Additionally, 63 (34%) parents maintained clinical levels of anxiety symptoms from T1 to T2, with 23 parents reporting lessoning symptoms, and 14 parents reporting increases in anxiety symtomology.

### Regression Results

#### Depression

A mediation model was tested with COVID-19 stress as a mediator between economic pressure and later depressive symptoms (Figure 1). Economic pressure was significant in predicting later depressive symptoms (*b* = 0.20, *p* = 0.013) and COVID-19 stress (*b* = 0.08, *p* < 0.001), such that greater economic pressure associated with increased depressive symptoms and COVID-19 stress. Likewise, COVID-19 stress significantly predicted later depressive symptoms (*b* = 0.90, *p* = 0.012), such that greater COVID-19 stress was strongly associated with increased depressive symptoms. Indirect effects in the model (*b* = 0.07, *SE* = 0.037, 95% CI [0.012, 0.156]) indicate that COVID-19 stress was a significant mediator of prior economic pressure on later depressive symptoms. Thus, COVID-19 stress contributes to economic pressure’s influence on parental depressive symptomology such that families experiencing higher economic pressure are likely to experience significantly greater COVID-19 related stress, which in turn increases their likelihood of experiencing substantial depressive symptomology later on.

Next, approach coping strategies was included as a moderator between COVID-19 stress and later depression symptoms in the previous model. Contrary to our predictions, approach coping strategies did not significantly moderate the association between COVID-19 worry and later depressive symptoms (*p* = 0.515).

Lastly, avoidant coping strategies were included as a moderator as in the previous analysis. The interaction effect of COVID-19 stress and parental avoidant coping strategies significantly predicted later depressive symptoms (*b* = 0.60, *p* = 0.009), and the indirect effect of the moderated mediation was significant (*b* = 0.08, *SE* = 0.042, 95% CI [0.005, 0.177]). Simple slopes were not significant for high (*p* = 0.672) or low (*p* = 0.735) reported use of avoidant coping. Results indicate that the interaction is significant, such that parents who use high avoidant strategies significantly differ in their association between COVID-19 stress and depressive symptomology compared to those with low strategies. Parents who utilized greater avoidant coping showed a greater association between COVID-19 stress and worsened depressive symptoms, while parents that utilized less avoidant coping showed a lower association between COVID-19 stress and later depressive symptoms. Results suggest that the interaction between COVID-19 stress, and avoidant coping exacerbates later depressive symptoms in parents.

#### Anxiety

A separate mediation model was tested with COVID-19 stress as a mediator between economic pressure and later anxiety symptoms (Figure 2). Economic pressure was significant in predicting later anxiety symptoms (*b* = 0.18, *p* = 0.005), and COVID-19 stress (*b* = 0.08, *p* < 0.001), such that greater economic pressure associated with greater anxiety symptoms and COVID-19 stress. COVID-19 stress also predicted increases in later anxiety symptoms (*b* = 0.72, *p* = 0.005). While economic pressure and COVID-19 stress were significant in predicting later anxiety symptoms; the indirect effect of the model suggests that COVID-19 stress was not a significant mediator (*b* = 0.06, *SE* = 0.034, 95% CI [−0.003, 0.136]). Thus, unlike the trends identified for depressive symptoms, the positive association between economic pressure and later anxiety symptoms was not significantly mediated by COVID-19 stress.

Approach coping strategies were then tested as a moderator between COVID-19 stress and later anxiety symptoms (Figure 3). Following recommendations from Holland et al. (2017), moderated mediation can be tested even when mediation is not significant alone, as a moderator may complete the model. The moderated mediation effect of COVID-19 stress and parental use of approach coping strategies significantly predicted later anxiety symptoms (*b* = 0.14, *p* = 0.016), and the indirect effect of the moderated mediation was significant (*b* = 0.07, *SE* = 0.034, 95% CI [0.009, 0.143]), thus completing the previous model. As shown in Figure 3, we plotted slopes for parents who reported low vs. high approach coping strategy utilization for the association between COVID-19 stress and later anxiety symptoms. The bar line in Figure 3 indicates moderate clinical cutoffs for anxiety symptoms based on the GAD-7 (summative score of 10; Spitzer et al., 2006a) to show that most parents reported moderate to high levels of anxiety symptoms. Simple slope analyses showed that there was a positive association between COVID-19 stress and later anxiety symptoms for parents who reported low (*t* = 2.25, *p* = 0.025) and high (*t* = 3.06, *p* = 0.003) use of approach coping strategies, though high use had a greater positive association between COVID-19 stress and later anxiety symptoms. These results suggest that the level of approach coping strategies utilized by parents modulates the association between their COVID-19 stress and later anxiety symptoms.

Finally, avoidant coping strategies was included as a moderator as in the previous set of analyses (Figure 4). The interaction for COVID-19 stress and parental avoidant coping strategies significantly predicted later anxiety symptoms (*b* = 0.83, *p* < 0.001), and the indirect effect of the moderated mediation was significant (*b* = 0.11, SE = 0.038, 95% CI [0.042, 0.193]). Simple slope analyses indicated a positive association between COVID-19 stress and later anxiety symptoms for parents reporting low (*t* = 3.02, *p* = 0.003) and high (*t* = 3.83, *p* < 0.001) use of avoidant coping strategies. COVID-19 stress and use of avoidant coping strategies were positively associated with greater later anxiety symptoms for parents. However, high avoidant coping had a stronger positive association with COVID-19 stress than did low use, such that when COVID-19 stress was greater parents with high avoidant coping reported greater later anxiety symptoms. Though parents with low use of avoidant coping and COVID-19 stress still reported clinically significant levels of anxiety symptoms. Results suggest interactions between COVID-19 stress and avoidant coping utilization exacerbate differences in later anxiety symptomology.

## Discussion

In this study, we evaluated the longitudinal impact of economic pressure and COVID-19 stress on internalizing mental health of parents with adolescent children, for whom little extant literature exists. Our results present three distinct findings which warrant attention and carry with them implications for family research and the FSM more broadly. Specifically, we found that event associated stress (e.g., pandemic related worry) mediated experiences of economic pressure and later depressive and anxious symptoms. Approach coping strategies were insufficient in managing anxiety symptomology during the pandemic, with greater use associated with higher reported anxiety symptoms later in the context of high COVID-19 stress. Finally, avoidant coping strategies also predicted later depression and anxiety symptoms, such that greater use of avoidant coping associated with greater reported mental health symptoms in the context of high COVID-19 stress, for both anxiety and depression. This study affirms that parents of adolescent’s face many of the same economic pressures and negative mental health effects as parents of younger children as they navigate the difficulties presented by the ongoing COVID-19 pandemic.

### Depressive Symptomology

According to the FSM, economic pressure influences family mental health (Masarik and Conger, 2017). In our model, COVID-19 stress functioned as a significant mediator of economic pressure and the development of depressive symptoms in parents of adolescents. Thus, the more parents worry about the safety and wellbeing of their family from the ongoing COVID-19 pandemic, the worse the depressive symptoms become. Our novel inclusion of COVID-19 stressors within the FSM adds contextual indicators of societal wellbeing that may not be fully captured in previous literature (e.g., Kämpfen et al., 2020; Prime et al., 2020). Thus, the prediction of parental depressive symptoms (as a specific aspect of mental health) can be improved by gathering context-specific lived experience about major events (e.g., pandemic specific worry).

In the context of daily functioning, depressive symptoms can wreak havoc on sleep, adaptability, and resilience (Hammen, 2005). In clinical settings, using approach coping strategies can reduce depressive symptoms by approximately 38%, although psychotherapy treatment has a greater effect (Cuijpers et al., 2009; Pozzi et al., 2015; SAMHSA, 2020). Despite our findings on the influence of COVID-19 worry on the development of depression symptoms, reported use of approach coping did not buffer this association. This non-significant effect of approach coping provides some important implications for the management of parental depression symptoms during pandemics. Although we assessed a wide array of common coping strategies (e.g., positive reframing and acceptance), it may be that the coping strategies measured have a low impact on managing pandemic related depressive symptoms because of the uniqueness and chronicity of the pandemic experience. Our finding may also reflect that reported use of coping strategies does not match their actual use, or the effectiveness of those strategies, when formally trained through psychotherapy (Cuijpers et al., 2009; Pozzi et al., 2015).

Avoidant coping strategies are consistent with worsening mental health in adult populations (Herman-Stabl et al., 1995). In this study, we found supporting evidence that avoidant coping strategies associated with worsening depressive symptoms over time for adult parents of adolescents. Specifically, greater use of avoidant coping associated with heightened depressive symptoms, while low use associated with lower depressive symptoms across levels of perceived COVID-19 stress. Our findings suggest that parental use of avoidant coping strategies worsened mental health during the COVID-19 pandemic. Future studies should more closely examine the role of coping in the management of depressive symptoms.

### Anxiety Symptomology

Similar to parental depressive symptoms, we found economic pressure and COVID-19 stress positively predict later anxiety symptoms in parents of adolescents. Interestingly, COVID-19 stress alone did not significantly mediate the relationship between economic pressure and later anxiety symptoms, though individual pathways were still significant. Prior work has found that worry (often conflated with stress) is linked to anxiety symptoms through shared emotions and reactions (Raes, 2010). Stress responses and reports are even used in testing for anxiety, and are often seen as possible symptoms of increasing anxiety (Spitzer et al., 2006a). In our study, COVID-19 stress was included as a mechanism for economic pressure to act upon later anxiety symptoms as it relates to societal experiences of stress and worry during the current pandemic. Through use of the FSM, we examined how inclusion of pandemic stress could better associate experiences of economic pressure and parental experiences of later anxiety symptoms (Masarik and Conger, 2017). Our findings suggest that while inclusion of COVID-19 stress does illustrate connections present, COVID-19 stress does not offer a significant pathway for economic pressure to influence later anxiety symptoms alone. Indicating that for these parents, processes outside of the scope of the current study are likely contributing to variability in their reports of anxiety symptoms. We believe that the inclusion of societal stressors in the model can be beneficial in describing key pathways and influences of economic pressure which lead to instances of later anxiety symptoms.

To examine potential buffering effects on later anxiety symptoms, we examined approach coping strategies on the pathway between COVID-19 stress and later anxiety symptoms. Approach coping had significant effects on the interaction between COVID-19 stress and later anxiety symptoms, such that the level of approach coping utilization associated with similar levels of COVID-19 stress and later anxiety symptoms. Greater use of approach coping was linked with higher levels of COVID-19 stress and later anxiety symptoms, while lower use of approach coping was associated with lower COVID-19 stress and later anxiety symptoms. Contrary to our hypothesis, heightened use of approach coping strategies did not diminish the association between COVID-19 stress and later anxiety symptoms, nor did it reduce later anxiety symptomology. Our findings indicate that parental use of approach coping strategies is dependent on levels of experienced stress during the pandemic. As coping strategies are taught and encouraged when individuals experience heightened levels of stress, it follows that use should decline when those experiences of stress decrease (Folkman and Lazarus, 1988). This reasoning aligns with prior findings on coping strategy use as those who are more likely to report greater use also report higher levels of experienced stress (Alosaimi et al., 2015; Roca et al., 2021). While reports of greater approach coping use are theorized to reduce later stress and anxiety, our findings indicate that there may be a conflictual link between approach coping strategies and later anxiety symptoms specifically that approach coping strategy use positively associates with later anxiety symptoms.

Continuing our examination of coping strategies, avoidant coping demonstrates an exacerbation of anxiety symptoms in the context of greater COVID-19 stress. As avoidant coping has been previously associated with worsening mental health (Herman-Stabl et al., 1995), our findings align with prior research to show a positive connection between greater use of avoidant coping and later anxiety symptoms. As parents have their own perceptions of how the pandemic is impacting them, their actions, such as avoidant coping, provide buffering effects for their later anxiety symptoms. Specifically, when parents report greater use of avoidant coping and high COVID-19 stress their later anxiety symptoms worsen, compared to low COVID-19 stress and high avoidant coping. However, lower use of avoidant coping may buffer the effect of high COVID-19 stress on later anxiety symptoms, such that low avoidant coping protects against exacerbating anxiety symptoms in parents. This suggests that perception of situational stress is indicative of later developed anxiety symptoms. Including contextual perceptions of events may then be critical for understanding parental mental health following stressful and unprecedented events.

In examining COVID-19 stress between economic pressure and later mental health in parents, our findings show that we can better understand mental health when accounting for both economic and societal stressors, such as the current pandemic. For both depressive and anxious symptoms, inclusion of coping strategies demonstrate the potential buffering and exacerbating effects when accounting for experienced stress. Future use of the FSM should include perception of societal stressors as they assist in predicting later mental health. Additionally, when examining parental mental health, careful consideration should be applied to the metrics used to gather this information. In our study, the GAD7 and PHQ9 were used to determine anxious and depressive symptom scores on a clinical level, though they are not sufficient alone in comprehending larger mental health clinical significance. Similarly, the use of coping strategies including adaptive and avoidant coping skills are by far not the only aspects of coping utilized in real world settings. Group members, such as family members, also influence and participate in stress management strategies (Kupst et al., 1995; Clark et al., 2014; Randall et al., 2016). Family coping, which includes the entire family as a unit, and dyadic coping, which includes two interacting members in a group, examine the ability of multiple members to work together to reduce and alleviate stress. Inclusion of more comprehensive measures of mental health and coping can help to identify mental health management and promote positive adaption in family populations. Future work should examine the effectiveness of various coping strategies over time and later mental health.

### Limitations and Future Directions

Strengths of this study include the sensitive period during which this data were collected and the focus on parents of adolescents who are often overlooked in parenting research. However, several limitations should be noted and considered for future work. First, pandemic data collected over a relatively short period may not be generalizable to experiences during other times throughout the pandemic. Our data spanned the 2020 fall school semester and so does not include reports from the initial onset of COVID-19, inclusion of the vaccines that were rolled out in early 2021, nor the continued spread and new variants (Roy et al., 2021; Vasireddy et al., 2021). For the current study, we focused on the sensitive period of the first in-person school semester since the pandemic was declared, which included many uncertainties for parents of adolescents regarding their children’s academic life and the spread of COVID-19. This study contextualized parents of adolescent’s experiences of stress following the onset of COVID-19 as it relates to their mental health across the fall 2020 academic semester. Future work will benefit from inclusion of longitudinal data spanning from initial COVID-19 onset and beyond and will be of upmost importance to fully comprehend the effects of global systemic chronic stress on economic pressure and mental health. Second, while our sample was reflective of the geographic area in which it was collected, it represented a strongly homogenized group with relatively high average income and educational attainment. Thus, many of these families may have benefited from more stable employment and greater access to resources than many families. To understand the full range of effects COVID-19 has on family functioning, future research should prioritize examining more diverse families. Particularly, those with less access to support and at higher risk of experiencing prolonged economic pressure and stress resulting from COVID-19 (e.g., parents employed in service-based industries, with lower educational attainment, or who lack resources to balance work and childcare needs). Importantly, our study examined parents of adolescents, who are often overlooked in parenting research, though these parents report increased stress and conflict with their adolescent children when compared to those with young children. Third, although we examined mental health symptoms for anxiety (GAD7) and depression (PHQ9), individuals were not evaluated formally for meeting diagnostic criteria for any disorders. Future work should consider including diagnosis of mental illness as it relates to improving our understanding of economic pressures and later experiences of mental health. Finally, though we examined parental reports on mental health, the relationship parents shared with their adolescent were not examined. The shared relationship between parent and child has been known to influence mental health reports (Gittleman et al., 1998; Cooke et al., 2019), and is expected to influence family mental health during the time of COVID-19. Future work should consider how shared relationship quality may impact reports of mental health during the pandemic, and in further exploration of the FSM.

To conclude, this study examined the mental health of parents of adolescents through the FSM and included COVID-19 stress as a mediator of economic pressure and later mental health. COVID-19 stress acts as a mechanism by which economic pressure can negatively impact later anxiety and depression symptomology. Approach coping was also examined as a buffer between COVID-19 stress and later anxiety experiences. Our findings indicate that the level of COVID-19 stress experienced relates to the level of coping skills used and later reported anxiety symptoms. The mental health of parents of adolescents requires greater attention given the lack of mental health resources available to parents during the pandemic (Jeffs et al., 2020; Usher et al., 2020). While parents are experiencing higher than average levels of anxiety and depression (Barzilay et al., 2020; Lee, 2020; Lee et al., 2021), utilization of approach coping skills was not found to reduce the clinical cutoff levels in our sample. These findings highlight that parents report utilizing approach coping strategies, but that current coping use may not be enough to offset negative mental health repercussions caused by the pandemic. Clinicians should prepare for treating an increasing number of parents of adolescents in their practices and ensure provision of additional skills that can be used to address clinical levels of anxiety and depressive symptoms in this population.

## Data Availability Statement

The raw data supporting the conclusions of this article will be made available by the authors, without undue reservation.

## Ethics Statement

The study involving human participants was approved by the Texas Tech University Institutional Review Board (IRB2020-607). Participants provided informed consent to participate in this study.

## Author Contributions

DM: data analyses and full manuscript write-up. CH: data analyses and manuscript revisions. PI: conceptual guidance and manuscript revisions. CR: design and funding of project, analysis guidance, and manuscript revisions. All authors contributed to the article and approved the submitted version.

## Conflict of Interest

The authors declare that the research was conducted in the absence of any commercial or financial relationships that could be construed as a potential conflict of interest.

## Publisher’s Note

All claims expressed in this article are solely those of the authors and do not necessarily represent those of their affiliated organizations, or those of the publisher, the editors and the reviewers. Any product that may be evaluated in this article, or claim that may be made by its manufacturer, is not guaranteed or endorsed by the publisher.
